# Quantized large language models in biomedical natural language processing: evaluation and recommendation

**DOI:** 10.1038/s44401-026-00152-6

**Published:** 2026-11-04

**Authors:** Zaifu Zhan, Shuang Zhou, Min Zeng, Yiran Song, Kai Yu, Meijia Song, Xiaoyi Chen, Jun Wang, Yu Hou, Rui Zhang

**Affiliations:** 1 Department of Electrical and Computer Engineering, University of Minnesota, Minneapolis, MN USA; 2 Division of Computational Health Sciences, Department of Surgery, University of Minnesota, Minneapolis, MN USA; 3 School of Nursing, University of Minnesota, Minneapolis, MN USA

**Keywords:** Computational biology and bioinformatics, Mathematics and computing

## Abstract

Large language models have demonstrated strong capabilities in biomedical natural language processing, yet their rapid growth in size and computational requirements creates a barrier to local deployment in privacy-sensitive settings. In this study, we evaluated the impact of quantization on 12 general-purpose and biomedical large language models across eight benchmark datasets covering named entity recognition, relation extraction, multi-label classification, and question answering. Using Transformers with BitsAndBytes, we found that 4-bit and 8-bit weight quantization substantially reduced peak GPU memory while often preserving task performance, although latency generally increased and degradation was task- and model-dependent. We further evaluated deployment-oriented AWQ, GPTQ, and FP8 checkpoints with vLLM on representative biomedical tasks and model scales. We also quantified invalid-output rates, prompt overhead, and few-shot memory effects. These results provide a systematic benchmark of quantization trade-offs for biomedical NLP and show that weight-memory reduction can make otherwise difficult-to-host large models feasible for local deployment on GPUs with sufficient VRAM, including high-memory consumer GPUs.

## Introduction

Large language models (LLMs) are increasingly used in biomedical natural language processing (NLP)^1–4^. General-purpose LLMs^5–7^ and domain-adapted biomedical models^8–11^ now perform well on many text-processing tasks. With prompt engineering^12–14^ and retrieval-augmented generation (RAG)^15–17^, these models can often be applied to new biomedical questions without task-specific retraining. These gains come with larger models and higher computational demands^18–20^. In domains where privacy requirements are less restrictive, cloud-based systems can provide access to large-scale models. In biomedical settings, however, patient confidentiality and data security often limit cloud deployment^21–23^. Local inference may therefore be preferred or required. This creates a practical challenge: deploying high-performing models under limited local computing resources while maintaining task performance.

This challenge sits within a broader shift in text classification. Earlier systems relied on rule-based, statistical, and supervised feature-based methods. Transfer learning and generative modeling then moved the field toward BERT- and GPT-style approaches^24^. Biomedical LLM prompting extends this trajectory, but it does not remove the value of smaller task-specific systems. This distinction is important for our multi-label classification experiments, where a generative model is used to solve a task that can also be formulated as conventional classification.

**Fig. 1:**
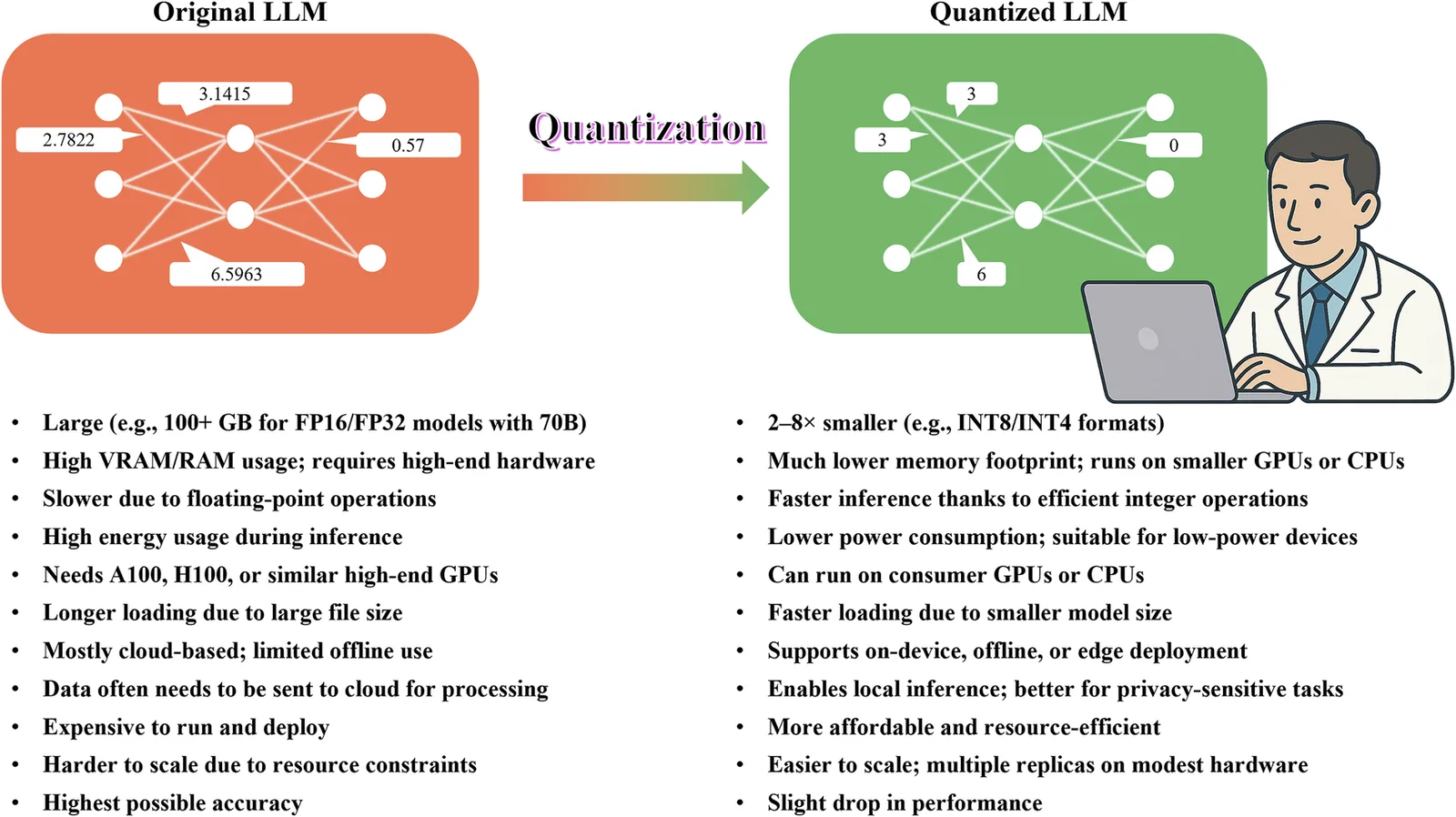
Comparison between default LLMs and their quantized counterparts.

One approach to this challenge is model quantization^25,26^, which reduces the precision of model weights, typically from 32- or 16-bit floating point representations to 8-bit or even lower formats, as shown in Fig. 1. By compressing the model in this way, quantization can decrease memory use and computational workload during inference. This can support local deployment on less powerful hardware, including consumer GPUs or edge devices, when those systems have enough memory for the selected model^27,28^. These improvements are particularly relevant in biomedical settings, where data often cannot be transferred off-site and high-performance GPUs may not be readily available. For this reason, quantization is an important tool for deploying LLMs in resource-constrained environments^29,30^.

Despite the growing interest in model quantization, most existing studies have primarily focused on general-domain NLP tasks and benchmarks. For example, Liu et al.^31^ and Li et al.^27^ conducted extensive evaluations of quantized LLMs on a variety of general-purpose NLP tasks, demonstrating their efficiency and competitive performance. Jin et al.^28^ provided a comprehensive analysis of instruction tuning for quantized LLMs and offered practical recommendations for deployment. Additionally, Li et al.^32^ explored the reasoning capabilities of quantized LLMs in mathematical tasks, while Marchisio et al.^33^ investigated quantization strategies for multimodal models. Further studies have examined the combination of quantization with low-rank adaptation techniques such as LoRA for fine-tuning, as seen in works by Xu et al.^34^ and Zhou et al.^35^. In contrast, the impact of quantized LLMs on biomedical tasks remains largely unexplored. Biomedical text presents unique challenges compared to general-domain corpora, including domain-specific terminology, more complex syntax, and highly specialized semantics. These differences mean that conclusions drawn from general-domain evaluations may not directly transfer to biomedical applications. Therefore, it is crucial to empirically assess the effectiveness, robustness, and trade-offs of quantized LLMs within the biomedical NLP landscape.

To address this gap, we evaluate quantized large language models on biomedical NLP tasks. Our goal is to characterize the practical trade-offs of using quantized LLMs in privacy-sensitive, resource-constrained biomedical environments and to provide evidence for researchers and system builders. Specifically, we conduct experiments across a variety of biomedical tasks, evaluating model behavior under different quantization settings, model architectures, and model sizes. The main experiment uses 4-bit and 8-bit BitsAndBytes quantization in Transformers, while an additional serving-oriented experiment evaluates AWQ^36^, GPTQ^37^, and FP8^38^ checkpoints with vLLM^39^. We also investigate few-shot learning, chain-of-thought prompting, self-consistency prompting, and invalid-output rates. By exploring these dimensions, we aim to establish a clearer understanding of the trade-offs between memory, latency, throughput, output validity, and task performance. Because memory capacity is often the primary barrier to local deployment, the peak-memory results show how quantization can enable large-model inference on GPUs with sufficient VRAM, including high-memory consumer GPUs.

## Results

This section summarizes how quantization affects task performance, GPU memory use, response latency, output validity, model size, and prompting strategy.

### Quantized LLMs on MLC, NER, RE, QA tasks

**Fig. 2:**
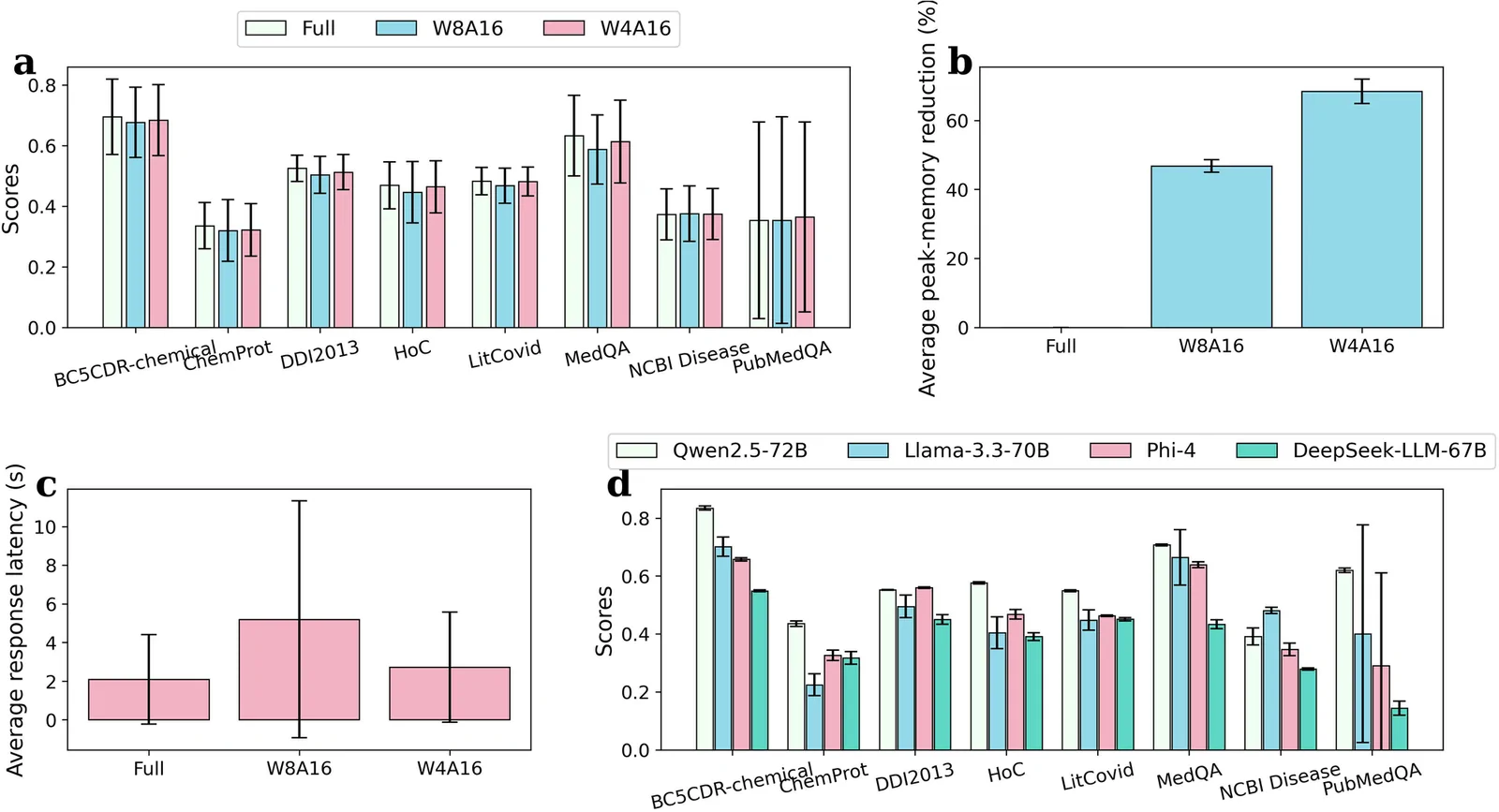
Quantitative comparison of quantization settings and general-purpose LLMs. **a** Mean task-specific score for full-precision, W8A16, and W4A16 models across eight biomedical datasets. Scores are averaged across Qwen2.5-72B, Llama-3.3-70B, Phi-4, and DeepSeek-LLM-67B. **b** Mean reduction in peak GPU memory relative to full precision. **c** Mean response latency under each precision setting. **d** Mean task-specific score of each model across the three precision settings. Scores represent macro F1 for multi-label classification and relation extraction, entity F1 for named entity recognition, and accuracy for question answering. Bars show means, and error bars show standard deviations. W8A16 denotes 8-bit weights with 16-bit activations, and W4A16 denotes 4-bit weights with 16-bit activations.

Table 1 summarizes the performance of four LLMs across eight datasets covering four distinct tasks, under three levels of numerical precision. Fig. 2 presents a comparison of model performance, average peak-memory reduction relative to full precision, and average response latency under different quantization settings. It also includes a performance comparison across different LLMs. The results show that quantized models often achieve large peak-memory reductions with limited average performance loss, but the effects vary across tasks and models. Quantization also increases response latency in the Transformers/BitsAndBytes setting. Among the four general-purpose models evaluated, Qwen2.5-72B shows the strongest overall performance. Table 2 further summarizes task-level sensitivity relative to full precision. Across task groups, 4-bit quantization usually gives the largest memory reduction with small mean score changes, whereas 8-bit loading shows larger latency ratios and more variable score changes. Table 3 reports invalid-output and repetition rates. The full-precision, 8-bit, and 4-bit settings all produce a nonzero fraction of malformed or repetitive outputs, indicating that output validity should be monitored in addition to aggregate task scores.

**Table 1:** Performance and efficiency comparison of full-precision and quantized LLMs (W8A16: 8-bit weights with 16-bit activations; W4A16: 4-bit weights with 16-bit activations) across biomedical NLP tasks, including multi-label classification, named entity recognition, relation extraction, and question answering

Dataset—Task / Model	Metric	Full precision			W8A16			W4A16		
		Mem	Late	Score	Mem	Late	Score	Mem	Late	Score
**HoC—Multi-label document classification**										
DeepSeek-LLM-67B	Macro F1	126.5	4.57	0.391	65.61	10.4	0.404	36.87	6.84	0.377
Llama-3.3-70B	Macro F1	132.37	3.74	0.45	68.85	11.4	0.343	39.38	4.87	0.419
Phi-4	Macro F1	28.41	1.05	0.459	16.08	2.27	0.458	10.72	1.45	0.487
Qwen2.5-72B	Macro F1	136.41	2.75	0.576	72.58	6.83	0.581	41.0	3.48	0.573
**LitCovid—Multi-label document classification**										
DeepSeek-LLM-67B	Macro F1	127.15	1.96	0.444	66.37	4.73	0.452	37.41	2.36	0.456
Llama-3.3-70B	Macro F1	133.08	1.47	0.481	69.7	3.55	0.41	39.95	2.0	0.452
Phi-4	Macro F1	29.2	0.47	0.461	16.91	1.02	0.462	11.36	0.67	0.465
Qwen2.5-72B	Macro F1	137.13	1.27	0.546	73.46	2.97	0.548	41.6	1.67	0.553
**BC5CDR-chemical—Named entity recognition**										
DeepSeek-LLM-67B	Entity F1	125.97	6.48	0.547	64.92	16.58	0.547	36.41	8.95	0.552
Llama-3.3-70B	Entity F1	131.87	6.52	0.734	68.21	15.77	0.668	38.94	7.15	0.7
Phi-4	Entity F1	27.78	1.97	0.656	15.23	4.66	0.663	10.23	2.82	0.653
Qwen2.5-72B	Entity F1	135.87	6.8	0.842	71.62	17.79	0.828	40.56	7.73	0.833
**NCBI Disease—Named entity recognition**										
DeepSeek-LLM-67B	Entity F1	125.89	6.22	0.279	64.82	15.95	0.275	36.34	9.09	0.282
Llama-3.3-70B	Entity F1	131.77	6.52	0.469	68.12	17.31	0.488	38.88	7.56	0.486
Phi-4	Entity F1	27.68	1.96	0.332	15.1	4.74	0.336	10.16	2.93	0.372
Qwen2.5-72B	Entity F1	135.78	6.94	0.413	71.49	18.76	0.402	40.49	7.86	0.357
**ChemProt—Relation extraction**										
DeepSeek-LLM-67B	Macro F1	126.43	1.01	0.324	65.54	2.52	0.334	36.81	1.52	0.294
Llama-3.3-70B	Macro F1	132.3	0.82	0.249	68.73	1.64	0.181	39.3	1.09	0.243
Phi-4	Macro F1	28.27	0.37	0.336	15.82	0.62	0.338	10.62	0.37	0.305
Qwen2.5-72B	Macro F1	136.33	0.74	0.435	72.53	1.67	0.427	40.95	1.03	0.446
**DDI2013—Relation extraction**										
DeepSeek-LLM-67B	Macro F1	126.19	0.87	0.464	65.29	2.03	0.452	36.65	1.3	0.432
Llama-3.3-70B	Macro F1	132.03	0.83	0.524	68.48	1.51	0.45	39.14	1.12	0.51
Phi-4	Macro F1	28.03	0.25	0.56	15.59	0.5	0.562	10.43	0.38	0.557
Qwen2.5-72B	Macro F1	136.09	0.82	0.552	72.24	1.86	0.55	40.78	1.18	0.553
**MedQA—Question answering**										
DeepSeek-LLM-67B	Accuracy	126.75	0.75	0.441	65.89	1.77	0.445	37.08	1.07	0.416
Llama-3.3-70B	Accuracy	132.63	0.5	0.732	69.15	0.88	0.555	39.58	0.77	0.705
Phi-4	Accuracy	28.67	0.25	0.648	16.29	0.53	0.64	10.94	0.39	0.628
Qwen2.5-72B	Accuracy	136.7	0.45	0.71	72.76	0.92	0.709	41.26	0.73	0.704
**PubMedQA—Question answering**										
DeepSeek-LLM-67B	Accuracy	126.44	0.7	0.166	65.5	1.58	0.146	36.82	1.1	0.118
Llama-3.3-70B	Accuracy	132.34	0.63	0.706	68.78	1.07	0.758	39.33	0.89	0.756
Phi-4	Accuracy	28.31	0.2	0.61	15.87	0.4	0.532	10.66	0.34	0.602
Qwen2.5-72B	Accuracy	136.37	0.48	0.616	72.46	0.92	0.614	40.97	0.8	0.628

Reported metrics include memory usage (Mem, in GB), inference latency (Late, in seconds), and task-specific scores (F1 / Accuracy).

**Table 2:** Task-level sensitivity of BitsAndBytes quantization relative to full precision

Task	Quant.	Mean Δ	Median Δ	Mem. ratio	Lat. ratio
MLC	4bit	−0.011	−0.010	0.320	1.312
	8bit	−0.032	−0.001	0.544	2.647
NER	4bit	−0.015	−0.010	0.316	1.338
	8bit	−0.054	−0.008	0.534	2.864
QA	4bit	−0.001	−0.011	0.325	1.541
	8bit	−0.020	−0.004	0.542	2.235
RE	4bit	−0.009	−0.007	0.318	1.420
	8bit	−0.017	−0.002	0.540	2.431

Values summarize the original experiment matrix. Mean Δ and median Δ denote score changes relative to full precision. Memory and latency are reported as ratios to full precision; lower memory ratios indicate larger memory savings.

**Table 3:** Mean invalid-output and repetition rates in the main experiment matrix

Quantization	Invalid output rate	Repetition rate
Full precision	11.3%	0.18%
8bit	13.9%	0.23%
4bit	12.0%	0.21%

Invalid outputs were identified from generated responses before metric computation using task-specific parsers and a lightweight repetition scan.

### Performance of domain-specific quantized LLMs across biomedical NLP tasks

**Fig. 3:**
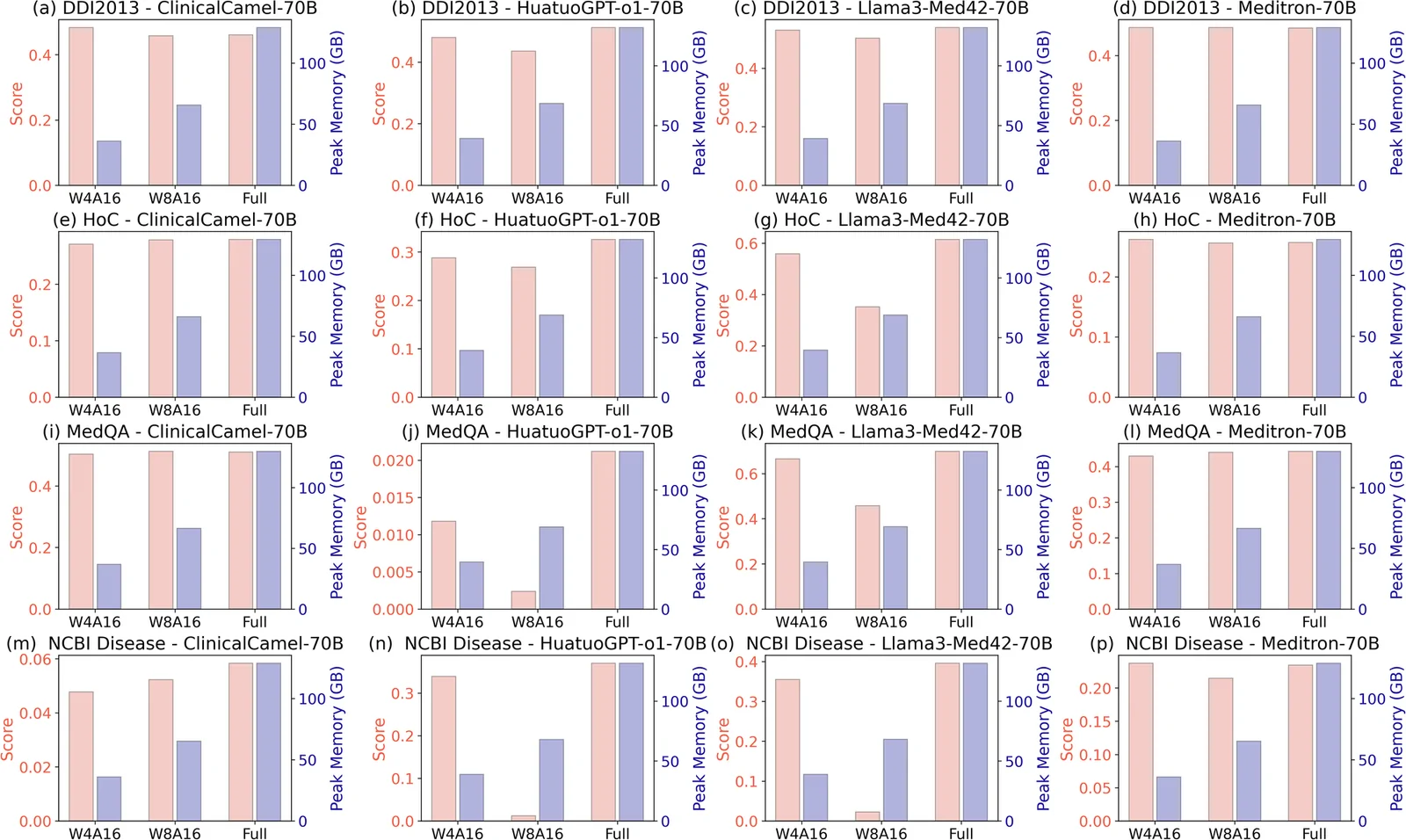
Performance and peak GPU memory of domain-specific biomedical LLMs under different quantization settings. **a**–**d** DDI2013 results for ClinicalCamel-70B, HuatuoGPT-o1-70B, Llama3-Med42-70B, and Meditron-70B, respectively. **e**–**h** HoC results for the same four models. **i**–**l** MedQA results for the same four models. **m**–**p** NCBI Disease results for the same four models. In each panel, the left axis and pink bars show the task-specific score, while the right axis and purple bars show peak GPU memory in GB. Scores represent macro F1 for DDI2013 and HoC, accuracy for MedQA, and entity F1 for NCBI Disease. W8A16 denotes 8-bit weights with 16-bit activations, and W4A16 denotes 4-bit weights with 16-bit activations.

Many biomedical LLMs are adapted with domain-specific text or instruction data. We therefore evaluated whether quantization preserves performance in these domain-specific models. Fig. 3 compares the performance and memory consumption of the ClinicalCamel-70B, HuatuoGPT-o1-70B, Llama3-Med42-70B, and Meditron-70B models on the DDI2013, HoC, MedQA, and NCBI Disease datasets. As shown in the figure, quantized LLMs exhibit a significant reduction in GPU memory requirements across all cases. Notably, for the DDI2013 and HoC datasets, quantization has minimal impact on model performance, especially when quantized to 4 bits. However, in some cases, HuatuoGPT-o1-70B and Llama3-Med42-70B show substantial performance degradation when quantized to 8 bits. Our observations indicate that the instruction-following capability of HuatuoGPT-o1-70B and Llama3-Med42-70B deteriorates under 8-bit quantization, with the models occasionally producing repetitive and semantically nonsensical outputs.

### Additional quantization methods with vLLM serving

To evaluate deployment-oriented quantization beyond BitsAndBytes, we conducted a serving-oriented experiment using vLLM^39^ with three widely used checkpoint families: AWQ, GPTQ, and FP8. vLLM reserves GPU memory for serving and for the key-value (KV) cache, which stores attention information during generation. Because this memory behavior differs from the Transformers/BitsAndBytes setting, we treat these experiments as throughput and accuracy evaluations rather than direct memory comparisons with Table 1. Table 4 summarizes representative results on HoC, NCBI Disease, DDI2013, and MedQA with Qwen2.5-7B and Qwen2.5-32B. The table reports task scores and generated-token throughput for AWQ, GPTQ, and FP8 under the same vLLM workflow. Across datasets, no single quantization method performed best on all tasks, reinforcing the need to evaluate quantization jointly with model scale, task type, and inference engine.

**Table 4:** Additional serving-oriented quantization evaluation with vLLM^39^

Dataset	Model	AWQ score	AWQ tok/s	GPTQ score	GPTQ tok/s	FP8 score	FP8 tok/s
DDI2013	Qwen2.5-32B	0.535	2717.0	0.542	2900.7	0.536	1873.5
	Qwen2.5-7B	0.483	11,671.1	0.487	12,148.6	0.505	11,397.7
HoC	Qwen2.5-32B	0.350	2329.6	0.524	2427.7	0.403	1238.9
	Qwen2.5-7B	0.451	8538.3	0.456	8803.4	0.457	7915.6
MedQA	Qwen2.5-32B	0.665	2707.0	0.653	2856.6	0.673	2381.2
	Qwen2.5-7B	0.518	11,302.2	0.504	11,849.5	0.543	11,015.1
NCBI Disease	Qwen2.5-32B	0.395	1352.4	0.360	1265.3	0.367	181.8
	Qwen2.5-7B	0.283	3991.9	0.296	4154.5	0.337	3487.1

Scores are macro/entity F1 for DDI2013, HoC, and NCBI Disease, and accuracy for MedQA. Throughput was measured with batched vLLM inference on NVIDIA A100 GPUs; it should not be directly compared with the Transformers/BitsAndBytes memory measurements in Table 1.

### Quantized LLMs with different size

**Fig. 4:**
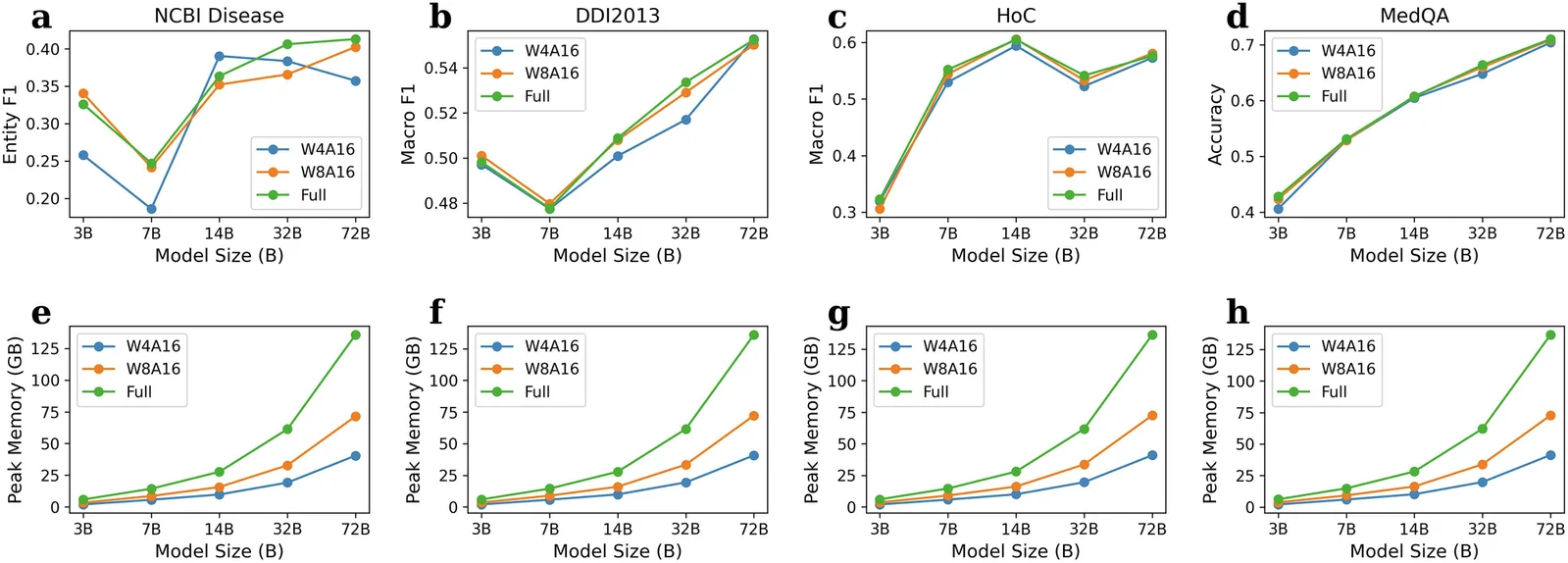
Effects of model size and quantization on the performance and peak GPU memory of Qwen2.5 models. **a**–**d** Task performance on NCBI Disease, DDI2013, HoC, and MedQA, respectively, for Qwen 2.5 models ranging from 3B to 72B parameters. **e**–**h** Corresponding peak GPU memory for NCBI Disease, DDI2013, HoC, and MedQA, respectively. Performance is measured using entity F1 for NCBI Disease, macro F1 for DDI2013 and HoC, and accuracy for MedQA. Lines represent full-precision, W8A16, and W4A16 configurations. W8A16 denotes 8-bit weights with 16-bit activations, and W4A16 denotes 4-bit weights with 16-bit activations.

The size of the model is a key factor to consider when deploying locally, as it directly impacts GPU memory requirements. Therefore, it is important to investigate how quantization techniques affect models of different sizes. Fig. 4 presents the performance (a–d) and memory usage (e–h) of Qwen2.5 models of various sizes under different quantization settings across four datasets: DDI2013, HoC, MedQA, and NCBI Disease. As the model size increases, memory usage grows approximately linearly. For the NCBI and HoC datasets, the performance of the 14B model has essentially plateaued, indicating saturation. In contrast, for the DDI and MedQA datasets, performance continues to improve with increasing model size. When comparing different quantization levels, the performance curves largely overlap, suggesting that quantized models can achieve comparable results to their full-precision counterparts. This highlights the effectiveness of quantization techniques in preserving model performance while reducing memory requirements.

### Quantized LLMs with few-shot learning

**Fig. 5:**
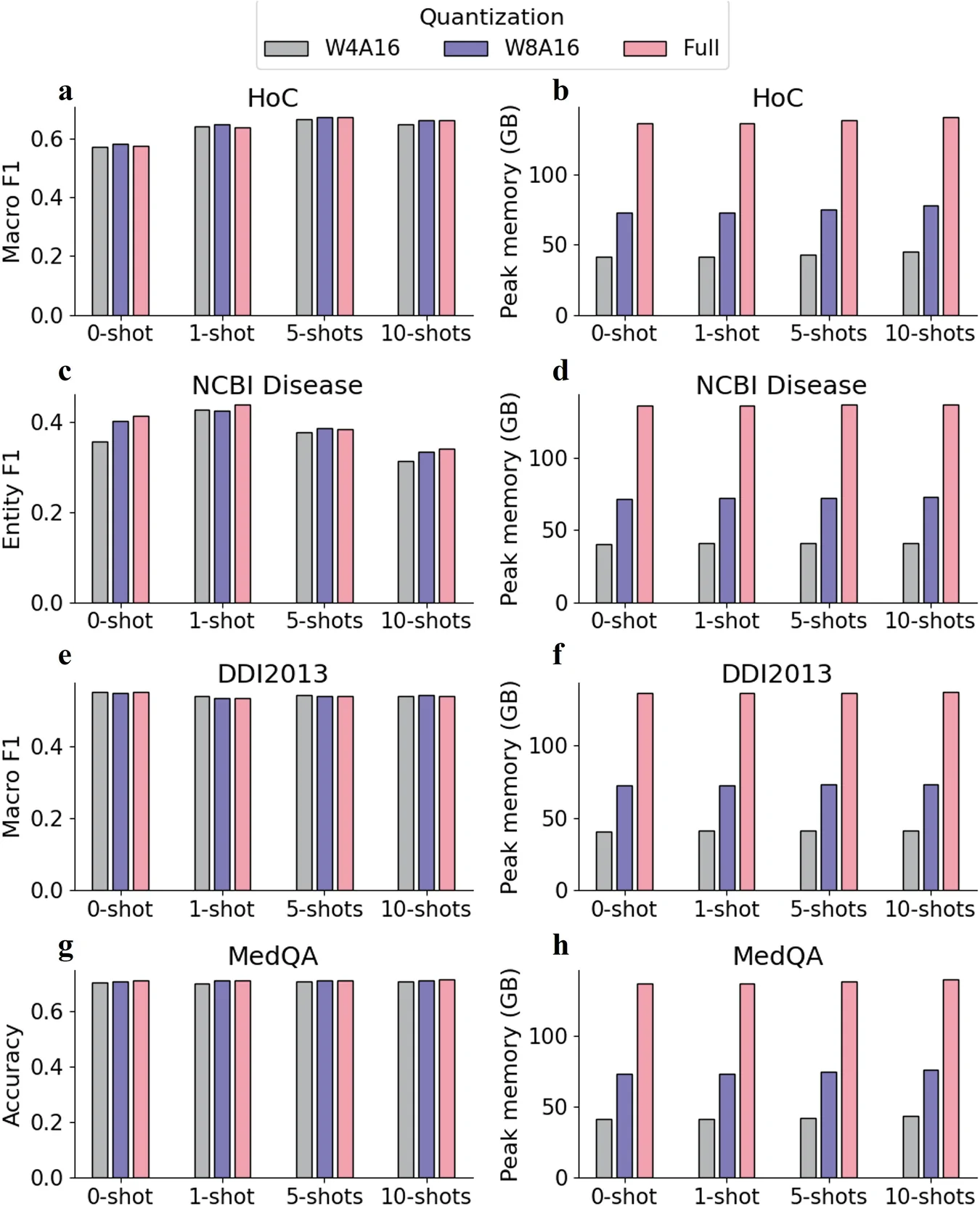
Effects of few-shot prompting on Qwen2.5-72B performance and peak GPU memory under different quantization settings. **a**, **c**, **e**, **g** Task performance with zero, one, five, and ten examples in the prompt for HoC, NCBI Disease, DDI2013, and MedQA, respectively. **b**, **d**, **f**, **h** Corresponding peak GPU memory for the same datasets and few-shot settings. Performance is measured using macro F1 for HoC and DDI2013, entity F1 for NCBI Disease, and accuracy for MedQA. Bars represent full-precision, W8A16, and W4A16 configurations. W8A16 denotes 8-bit weights with 16-bit activations, and W4A16 denotes 4-bit weights with 16-bit activations.

Few-shot learning can help LLMs perform a task by adding examples to the prompt. However, these examples make the prompt longer, increase generation cost, and enlarge the KV cache. This can raise GPU memory use even when the model weights are quantized. We therefore evaluated the memory and performance effects of few-shot prompting. As shown in Fig. 5, GPU memory usage increases with prompt length under different quantization settings.

### Prompting strategies on quantized LLMs

**Fig. 6:**
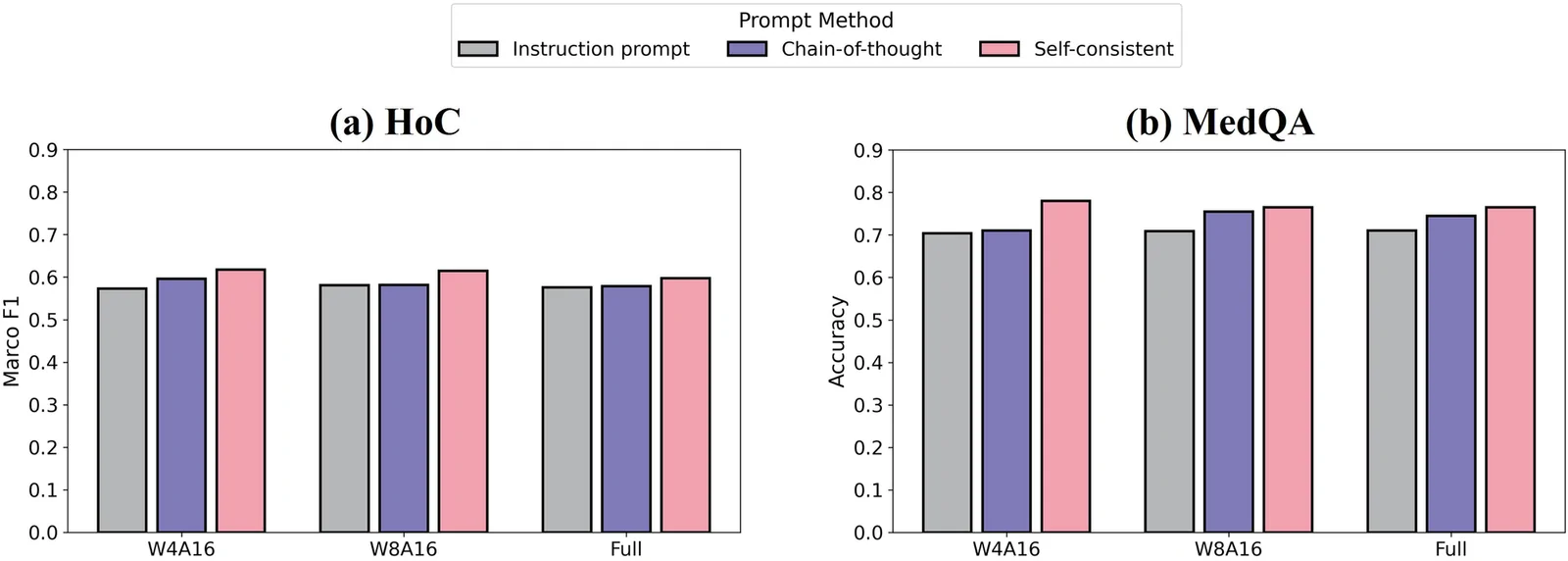
Effects of prompting strategy on the performance of full-precision and quantized Qwen2.5-72B. **a** Macro F1 on the HoC dataset. **b** Accuracy on the MedQA dataset. Each panel compares instruction prompting, chain-of-thought prompting, and self-consistent prompting under W4A16, W8A16, and full-precision configurations. W8A16 denotes 8-bit weights with 16-bit activations, and W4A16 denotes 4-bit weights with 16-bit activations.

Prompt design can strongly affect LLM performance without changing model weights. We therefore evaluated whether quantized models remain responsive to different prompting strategies. Fig. 6 illustrates the performance of three prompting approaches—instruction prompting, chain-of-thought (CoT) prompting, and self-consistent prompting. The results demonstrate that more sophisticated prompting methods yield substantial performance improvements. Notably, these trends are consistently observed across different quantization levels, indicating that quantized LLMs remain compatible with and responsive to advanced prompting techniques. However, the gains come with large computational costs. Table 5 shows that CoT and self-consistency substantially increase generated tokens and latency relative to instruction prompting, especially for MedQA. Thus, advanced prompting remains compatible with quantized models, but it shifts the deployment bottleneck from model weights toward generation length and KV-cache memory.

**Table 5:** Prompting overhead relative to instruction prompting for Qwen2.5-72B

Dataset	Prompt	Quant.	Score delta	Latency ratio	Token ratio
HoC	CoT	4bit	0.023	12.7	10.2
		8bit	0.001	17.4	11.4
		Full precision	0.003	16.1	11.1
	Self-consistency	4bit	0.045	12.9	10.3
		8bit	0.034	17.2	11.4
		Full precision	0.022	16.3	11.2
MedQA	CoT	4bit	0.006	76.8	28.8
		8bit	0.046	142.7	26.5
		Full precision	0.035	109.6	26.3
	Self-consistency	4bit	0.076	75.9	28.5
		8bit	0.056	144.9	26.6
		Full precision	0.055	110.0	26.4

More elaborate prompting can improve scores, but increases generated tokens and latency substantially.

## Discussion

This study evaluates LLM quantization for biomedical NLP across multiple task types, model families, model sizes, and prompting settings. The main Transformers/BitsAndBytes experiments show that 4-bit and 8-bit quantization can substantially reduce peak GPU memory, with 4-bit quantization giving the largest savings. However, quantization does not improve every deployment metric. Latency often increases in the Transformers/BitsAndBytes runtime, output validity varies by model and task, and some biomedical models show pronounced degradation under 8-bit loading. These findings support quantization as a useful deployment technique, but they also show that it must be validated for the target model, task, and runtime.

The task-level analysis helps explain why average performance can obscure important behavior. Relation extraction and multi-label classification were relatively stable in many cases, while named entity recognition and question answering showed more model-specific variation. The 8-bit anomalies observed for HuatuoGPT-o1-70B and Llama3-Med42-70B appear to be associated with poorer instruction following and repetitive or ill-formed outputs rather than a simple loss of biomedical knowledge. This distinction is important for deployment: downstream scores can degrade because the model fails to produce parseable task-formatted answers, even when its underlying language capability is not directly measured.

The AWQ, GPTQ, and FP8 experiments extend the benchmark beyond BitsAndBytes and show that the inference engine matters. We report these vLLM^39^ experiments as throughput and accuracy evidence, while keeping them separate from the Transformers/BitsAndBytes memory analysis. This separation is important because the inference engine, checkpoint format, batching behavior, and hardware kernels jointly affect runtime behavior.

Our few-shot and prompting experiments show another source of deployment cost. Quantization reduces weight memory, but longer prompts and longer generations increase KV-cache memory and latency. Chain-of-thought and self-consistency prompting can improve task scores, but the latency and token overhead are large, particularly for question answering. Therefore, selecting a quantized model is only one part of biomedical LLM deployment. Prompt design, maximum generation length, and batching policy can dominate end-to-end system behavior. KV-cache quantization is an important future direction for biomedical LLM deployment, especially for long-input tasks where context length rather than model weights becomes the dominant memory bottleneck. A rigorous evaluation of KV-cache quantization will likely require longer-context biomedical tasks than those emphasized in the current benchmark.

Quantized large LLMs should also be viewed alongside smaller or more specialized biomedical NLP systems. T5-Medical at SemEval-2024 Task 2 used T5 medical embeddings for natural language inference on clinical trial data^40^. This example does not replace the need to evaluate large biomedical LLM deployment, but it shows that task-specific systems may be more efficient when the clinical task is narrow and well defined.

Several limitations remain. First, all experiments were performed on NVIDIA A100 GPUs, and we did not have access to RTX or other consumer GPUs in this study. The memory results support the central feasibility claim: quantization can reduce model weight memory enough for large-model inference on GPUs with sufficient VRAM, including high-memory consumer GPUs. However, we did not directly benchmark different consumer GPU models. This limits hardware-specific claims about RTX throughput, latency, kernel behavior, and power use. Second, vLLM reserves memory for serving and KV cache, making its raw memory footprint difficult to compare directly with PyTorch peak allocation. Third, the benchmark datasets are public and have been widely used in biomedical NLP. They may have been included in the pretraining or instruction-tuning corpora of some evaluated LLMs, particularly recent general-purpose models. We therefore interpret absolute benchmark scores cautiously and do not treat them as direct evidence of clinical generalization. Fourth, although invalid-output analyses provide additional checks on result stability, the benchmark remains an offline evaluation rather than a prospective clinical validation study. Finally, the tested quantization methods cover common current approaches but do not exhaust the rapidly evolving quantization ecosystem.

**Fig. 7:**
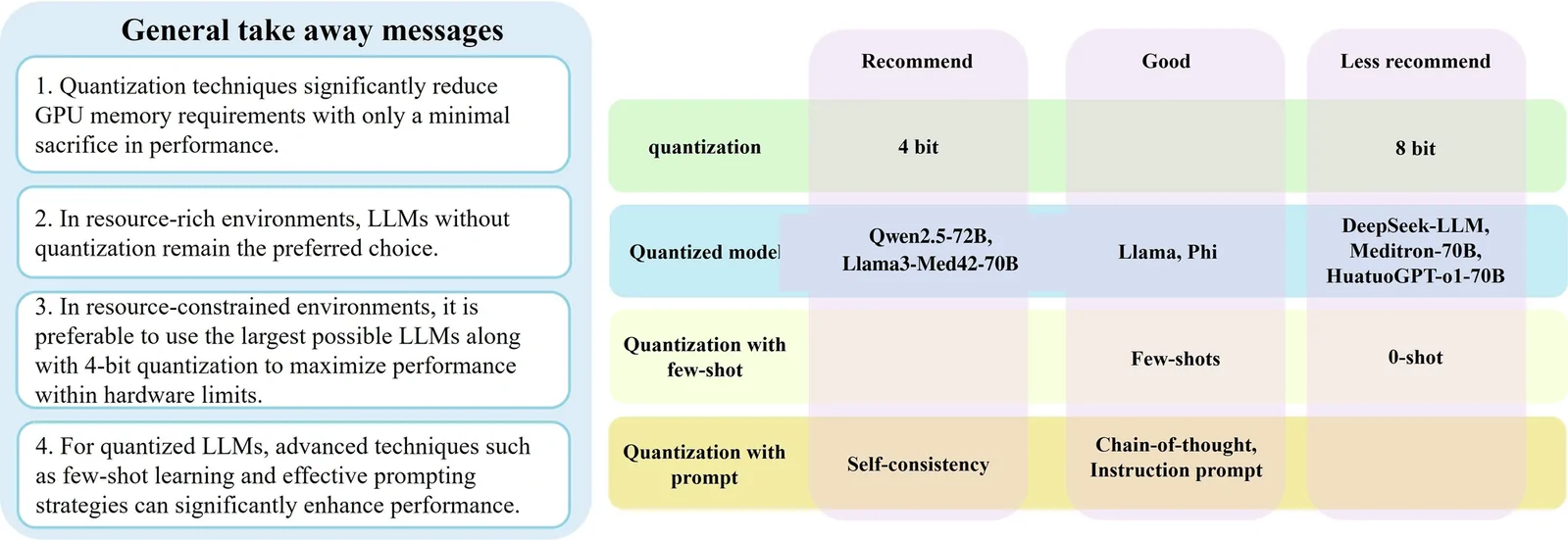
Recommendations for using quantized LLMs in biomedical natural language processing applications.

In conclusion, quantization can make large biomedical LLM experiments and local inference more resource efficient, especially when 4-bit weight quantization is acceptable for the target task. By reducing weight memory, quantization can allow large models that are otherwise difficult to host locally to run on high-memory consumer GPUs when VRAM is the primary bottleneck. The realized benefit still depends on model architecture, quantization method, inference engine, prompt length, and hardware-specific kernels. Fig. 7 summarizes practical recommendations for selecting quantized LLM configurations in biomedical NLP. We therefore recommend treating quantization as an empirically tested system choice rather than a universally reliable replacement for full-precision inference.

## Methods

### Datasets

To enhance the generalizability and practical value of our research, we selected four commonly studied tasks: named entity recognition (NER), relation extraction (RE), multi-label classification (MLC), and question answering (QA). Our dataset selection follows prior biomedical LLM benchmarking work^2^. For each task, we further chose two representative publicly available datasets: HoC^41^ and LitCovid^42^ for MLC, BC5CDR^43^ and NCBI^44^ for NER, ChemProt^45^ and DDI^46^ for RE, and MedQA-USMLE^47^ and PubMedQA^48^ for QA. Depending on the characteristics of each task, we applied task-specific evaluation metrics. All datasets are summarized in Table 6.

**Table 6:** Overview of the evaluation datasets and evaluation metrics.

Task	Dataset	size	Metric
Multi-label Classification	HoC	315	Macro F1
	LitCovid	2500	Macro F1
Named Entity Recognition	BC5CDR-chemical	4797	Entity-level F1
	NCBI-disease	940	Entity-level F1
Relation Extraction	ChemProt	16,943	Macro F1
	DDI2013	5761	Macro F1
Question Answering	MedQA-USMLE	1273	Accuracy
	PubMedQA	500	Accuracy

### LLMs

We evaluate a selection of representative large language models (LLMs) from both general-purpose and biomedical domains. Specifically, the general-purpose models include Llama-3.3-70B^49^, Qwen2.5-72B^50^, Phi-4^51^, and DeepSeek-LLM-67B^52^. For domain-specific models, we include ClinicalCamel-70B^53^, Llama3-Med42-70B^54^, HuatuoGPT-o1-70B^55^, and Meditron-70B^56^. To assess performance across different model scales, we also benchmark a range of Qwen2.5^50^ models with varying sizes: Qwen2.5-3B, Qwen2.5-7B, Qwen2.5-14B, Qwen2.5-32B, and Qwen2.5-72B.

### Quantization setup

To evaluate the impact of quantization on model performance, we apply a standard quantization setup using the Hugging Face Transformers library^57^. Specifically, we support both 4-bit and 8-bit quantization through the BitsAndBytes module^58,59^. For 4-bit quantization, we adopt the NF4 quantization scheme with float16 as the computation data type and enable double quantization to improve numerical stability. For 8-bit quantization, we use the default 8-bit loading configuration.

We additionally evaluate three checkpoint-level quantization methods using vLLM^39^: AWQ^36^, GPTQ^37^, and FP8^38^. These experiments use representative Qwen2.5-7B and Qwen2.5-32B checkpoints on HoC, NCBI Disease, DDI2013, and MedQA. We use vLLM for batched offline inference and report task score and generated-token throughput. We do not directly compare vLLM reserved memory with the Transformers/BitsAndBytes peak-memory measurements because vLLM preallocates memory for serving and KV cache.

### Prompt methods

To investigate the interaction between quantization techniques and prompting methods, we primarily adopt instruction prompts^12,60^ in our experiments. These prompts explicitly describe the task, expected output format, and provide the input to guide the model. Additionally, to explore the impact of different prompting strategies, we examine whether quantized models can still follow reasoning instructions and benefit from performance improvements when using approaches such as chain-of-thought^61^ and self-consistency prompting^62^.

### Experimental settings

Our code is implemented based on the Hugging Face *transformers* library^57^. All experiments are conducted primarily on four NVIDIA A100 GPUs, each with 40 GB of memory. We use the BitsAndBytes library^58,59^ to quantize LLMs, converting them from full precision to 8-bit or 4-bit integer formats. Despite the quantization, computations are internally performed in full precision, which helps maintain model accuracy during the forward pass. To optimize computational resources and reduce runtime, we dynamically adjust the output token length depending on the task. For instance, classification tasks typically restrict the output to around 50 tokens, whereas named entity recognition tasks may require generating complete sentences, allowing up to 200 tokens. All other model parameters follow the default configurations provided by the Hugging Face library.

### Latency, output validity, and statistical analysis

For latency measurement, we synchronized CUDA operations before and after generation in the Transformers experiments. We also computed generated-token throughput from total generated tokens and wall-clock duration. For each main result, we parsed model generations using the task-specific evaluation code and recorded invalid-output rates, including empty or unparseable outputs and lightweight repetition indicators. To quantify uncertainty, we computed nonparametric bootstrap confidence intervals for task scores from generated outputs. These analyses were used to assess whether apparent differences were consistent with output-level variability and to identify cases where score degradation was associated with malformed responses.

## Data Availability

The datasets used in this paper follow a previous benchmark study and are available at https://doi.org/10.5281/zenodo.14025500.

## Code availability

The code is publicly available at https://github.com/Learner4everrr/Benchmark_quantization.
